# The Housekeeping Gene Hypoxanthine Guanine Phosphoribosyltransferase (HPRT) Regulates Multiple Developmental and Metabolic Pathways of Murine Embryonic Stem Cell Neuronal Differentiation

**DOI:** 10.1371/journal.pone.0074967

**Published:** 2013-10-09

**Authors:** Tae Hyuk Kang, Yongjin Park, Joel S. Bader, Theodore Friedmann

**Affiliations:** 1 Department of Pediatrics and Rady Children's Hospital of San Diego, UCSD School of Medicine, La Jolla, California, United States of America; 2 Department of Biomedical Engineering, High-Throughput Biology Center, and Institute of Computational Medicine, Johns Hopkins University, Baltimore, Maryland, United States of America; Baylor College of Medicine, United States of America

## Abstract

The mechanisms by which mutations of the purinergic housekeeping gene hypoxanthine guanine phosphoribosyltransferase (HPRT) cause the severe neurodevelopmental Lesch Nyhan Disease (LND) are poorly understood. The best recognized neural consequences of HPRT deficiency are defective basal ganglia expression of the neurotransmitter dopamine (DA) and aberrant DA neuronal function. We have reported that HPRT deficiency leads to dysregulated expression of multiple DA-related developmental functions and cellular signaling defects in a variety of HPRT-deficient cells, including human induced pluripotent stem (iPS) cells. We now describe results of gene expression studies during neuronal differentiation of HPRT-deficient murine ESD3 embryonic stem cells and report that HPRT knockdown causes a marked switch from neuronal to glial gene expression and dysregulates expression of Sox2 and its regulator, genes vital for stem cell pluripotency and for the neuronal/glial cell fate decision. In addition, HPRT deficiency dysregulates many cellular functions controlling cell cycle and proliferation mechanisms, RNA metabolism, DNA replication and repair, replication stress, lysosome function, membrane trafficking, signaling pathway for platelet activation (SPPA) multiple neurotransmission systems and sphingolipid, sulfur and glycan metabolism. We propose that the neural aberrations of HPRT deficiency result from combinatorial effects of these multi-system metabolic errors. Since some of these aberrations are also found in forms of Alzheimer's and Huntington's disease, we predict that some of these systems defects play similar neuropathogenic roles in diverse neurodevelopmental and neurodegenerative diseases in common and may therefore provide new experimental opportunities for clarifying pathogenesis and for devising new potential therapeutic targets in developmental and genetic disease.

## Introduction

Lesch-Nyhan Disease (LND) is a monogenic neurodevelopmental disease caused by mutations in the X-linked gene encoding the purine salvage biosynthetic enzyme hypoxanthine-guanine phosphoribosyltransferase (HPRT) [1]. The clinical disorder is characterized by dystonia, choreoathetosis, cognitive deficits and self-injurious behavior, the hallmark feature of LND. The most prominent and well-recognized neurophysiological consequence of HPRT deficiency in the human central nervous system is dysfunction of basal ganglia dopaminergic (DA) neurons and defective development of DA signaling pathways [2]–[5] that in turn are thought at least partially to cause the aberrant neurological phenotype. The mechanisms connecting the defective purine pathways with neurological defects are not well understood, although most current models of LND pathogenesis assume that aberrant purine metabolism is the proximate cause of the neurological dysfunction through a direct effect of aberrant purine levels on early neural development or on neural function.

Recent studies in our laboratories have identified molecular neural dysregulatory mechanisms associated with HPRT deficiency that are likely to underlie defective neural development and aberrant function of dopaminergic and possibly other classes of neural cells. These findings point to a complex set of dysregulated functions and pathways that constitute a multi-systems set of pathogenic mechanisms responsible for this monogenic disease. Most relevant have been demonstrations of aberrant expression of key neuronal transcription factors, microRNA expression and defects in purinergic and other cellular signaling functions in a variety of mouse and human cell culture systems including human iPS cells. These defects have included aberrant canonical Wnt/b-catenin signaling and defective presenilin-1 expression [6], dysregulated expression of purinergic receptors with resulting aberrant expression of phospho-CREB and phospho-ERK signaling [7] and aberrant expression of microRNA expression [8]. These results have led to the surprising conclusion that the housekeeping HPRT gene serves not only to drive classical metabolic pathways, but also to regulate multiple key neurodevelopmental functions, a previously unrecognized role of a metabolic housekeeping gene. Still largely unstudied is the possibility that the HPRT protein carries out other purine-unrelated pleiotropic effects in other systems.

In the present study, we have taken advantage of a highly-efficient established protocol for dopaminergic neuronal differentiation of embryonic stem cells [9] and global transcriptome characterization via microarray and RNA-Seq methods to identify transcriptional aberrations in HPRT-knockdown murine ESD3 embryonic stem cells during neuronal differentiation in vitro. We have demonstrated that although wild type (WT) and HPRT-deficient murine ESD3 embryonic stem cells generate dopaminergic neuronal cells with approximately equal efficiency, HPRT-deficient cells show markedly aberrant patterns of expression of genes associated with dopaminergic neurogenesis and function. We performed microarray-based transcriptome analysis of cells at the pre-differentiation embryonic stem cell stage, at the partially differentiated SNM (spherical neural mass) stage [9] and at the fully differentiated neuronal stage. We also used RNA-Seq analysis for a detailed temporal characterization of the transcriptomes of WT and HPRT-knockdown ESD3 cells during the 14-day differentiation of SNM cells to fully differentiated neurons. Through these studies, we have discovered that HPRT deficiency causes aberrant regulation of multiple genetic and metabolic pathways including those that affect stem cell pluripotency and that drive cells in differentiation directions that involve a cell developmental fate choice to develop along neuronal or glial pathways. In addition, we have found multiple additional defects in HPRT-deficient cells, including aberrations related to cell cycle and proliferation mechanisms, RNA metabolism, DNA replication and repair, replication stress, lysosome function, membrane trafficking, signaling pathway for platelet activation (SPPA) multiple neurotransmission systems and sphingolipid, sulfur and glycan metabolism. We therefore hypothesize that the human HPRT-deficiency phenotype may constitute a “systems” disorder that produces broad and powerful dysregulation of many aspects of neurodevelopment by mechanisms possibly stemming from dysregulated pluripotency or inappropriate stem cell entry into aberrant differentiation pathways. If correct, such broad developmental mechanisms represent unexpected functions for a metabolic housekeeping gene.

## Results

### Preparation of control and HPRT knockdown ESD3 cells

**Figure 1:**
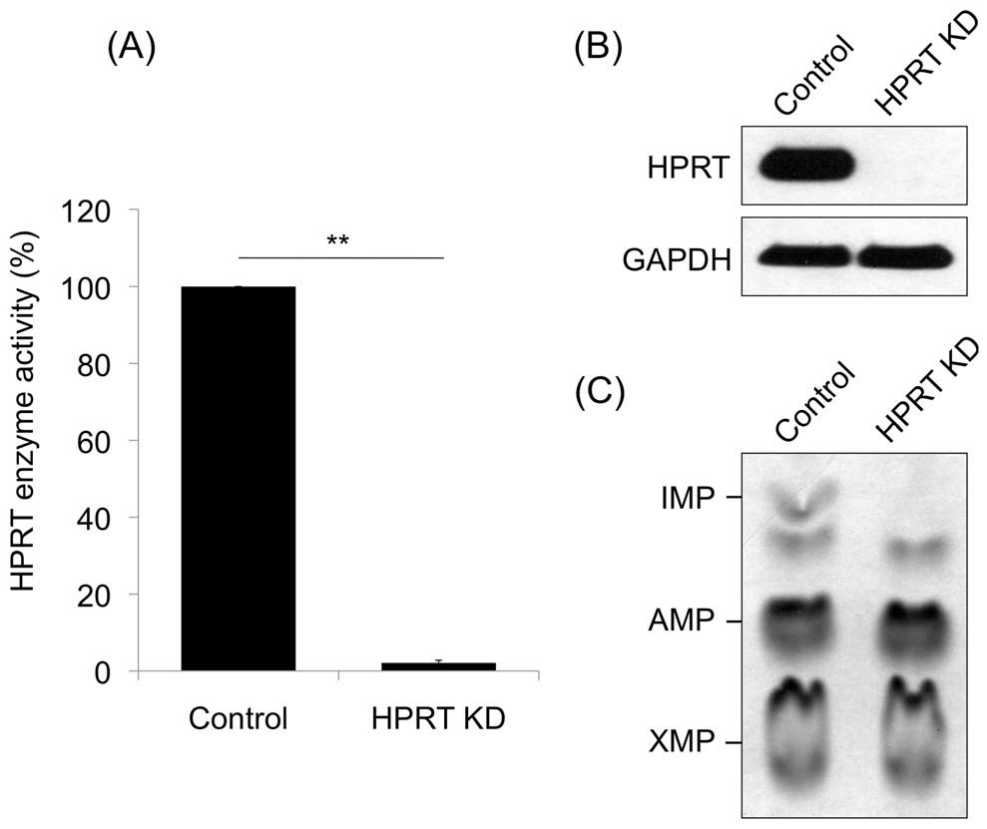
HPRT knockdown in murine ESD3 cells. (A) Reduction of HPRT mRNA expression as assayed by qPCR; (B) reduction of immunoreactive HPRT protein as determined by Western blot analysis; (C) reduction of HPRT enzymatic activity as measured by absent detectable IMP production as detected by autoradiography of a thin layer chromatography of cell lysate (6).

The HPRT mRNA level in HPRT knockdown cells was down-regulated by approximately 98% compared with WT cells (Figure 1A) and HPRT protein expression was markedly reduced in HPRT knockdown cells (Figure 1B). The enzymatic activity of HPRT was also significantly reduced by a comparable amount in HPRT knockdown cells as measured by a Western blotting assay (Figure 1C). Wild type cells continued to express HPRT at high levels throughout the differentiation process, although the level of expression at the SNM and neuronal stages was somewhat reduced compared with the ES stage. The knockdown cells, as expected, showed very low expression throughout differentiation. (data not shown).

### Neuronal differentiation

**Figure 2:**
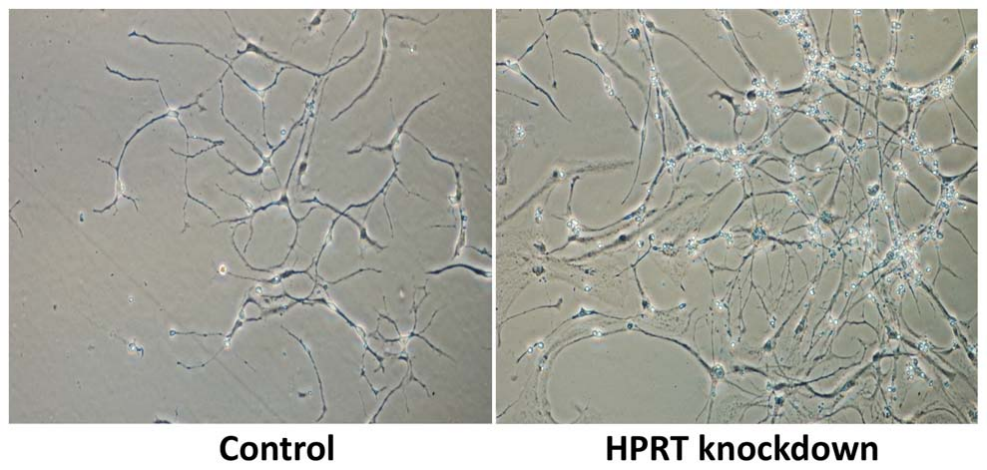
Neuron-like cells generated from WT control and HPRT-knockdown ESD3 cells.

WT and HPRT knockdown ESD3 cells both undergo similarly efficient degrees of differentiation to the DA neuronal phenotype, as described previously [9]. Fig. 2 illustrates typical neuronal morphology of a sparse field of cells differentiated from SNM derived from WT and HPRT-knockdown ESD3 cells following the full 14-day differentiation process. The WT and knockdown cells produced cells with similar typical neuronal morphology.

**Figure 3:**
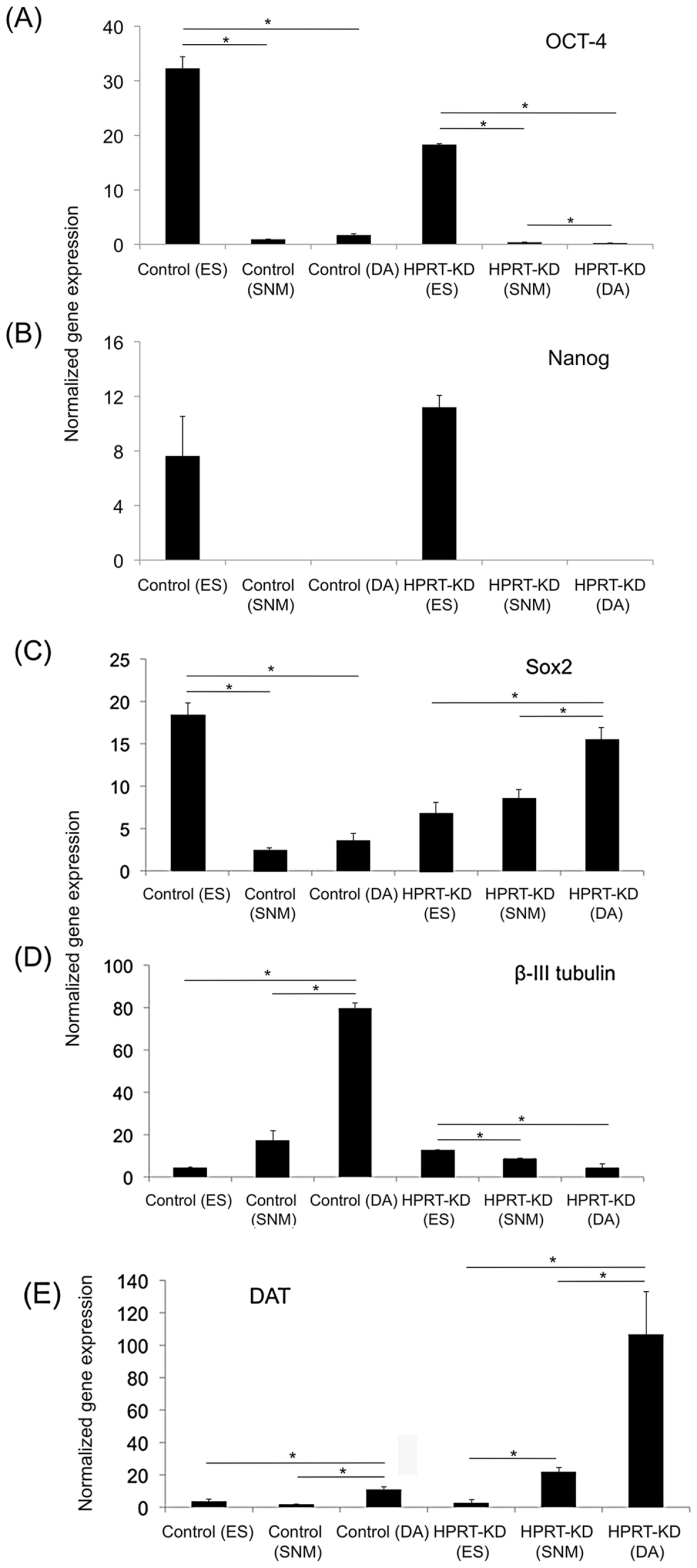
Regulation of pluripotency markers at the embryonic stem cell, SNM and neuronal (DA) stages of neuronal differentiation of WT control and HPRT-knockdown ESD3 cells, as measured by qPCR analysis. (A) Expression of Oct-4, showing expected down regulation; (B) expression of Nanog during differentiation; (C) expression of Sox2 during differentiation. Control cells show the expected down regulation at the SNM and DA stages but HPRT-knockdown cells show up-regulated expression during both SNM and DA stages of differentiation; (D) expression of the neuronal marker β-III tubulin showing the expected up-regulation in control cells, but reduced expression during differentiation of HPRT knockdown cells; (E) up-regulation of the dopaminergic neuronal marker DAT in control cells and HPRT-knockdown cells.

For further evaluation of the molecular events accompanying neuronal differentiation, we analyzed mRNA expression levels of pluripotency and neuronal markers by qPCR through the differentiation process at the embryonic, spherical neural mass (SNM) and fully differentiated neuronal stages. As expected, the pluripotency markers OCT-4 and Nanog became significantly down-regulated in both WT and knockdown cells as they differentiated from the embryonic stage through the SNM stage to the fully differentiated neuronal stage (Figure 3A and B). However, while WT cells also showed efficient down-regulation of the pluripotency and neuronal/glial developmental marker Sox2 during differentiation, expression of Sox2 not only failed to down-regulate in HPRT-knockdown cells but instead was up-regulated at the SNM stage and even further markedly up-regulated at the neuronal stage (Figure 3C). As expected, the neuronal marker β-III tubulin showed marked up-regulation in WT cells, particularly in the differentiation step from SNM to the neuronal phenotype. However, the HPRT-knockdown cells failed completely to up-regulate β-III tubulin expression at either the SNM or DA neuronal stage (Figure 3D) as did the WT cells. In contrast, the neuronal marker directly relevant to the dopaminergic system; i.e., dopamine transporter (DAT), showed modest up-regulation in WT cells at the neuronal stage (Figure 3E) while the HPRT-knockdown cells showed an earlier and much more robust up-regulation at the fully differentiated neuronal stage (Figure 3E). Interestingly, regulation of TH was not as clearly or consistently affected by HPRT deficiency. The results for TH transcription were inconsistent and un-interpretable. Briefly, both the wild type and knockdown cells demonstrate alternating levels of increased and decreased expression during the differentiation process (data not shown). We therefore have not yet been able to identify a reproducible regulatory role of HPRT in expression of TH.

**Figure 4:**
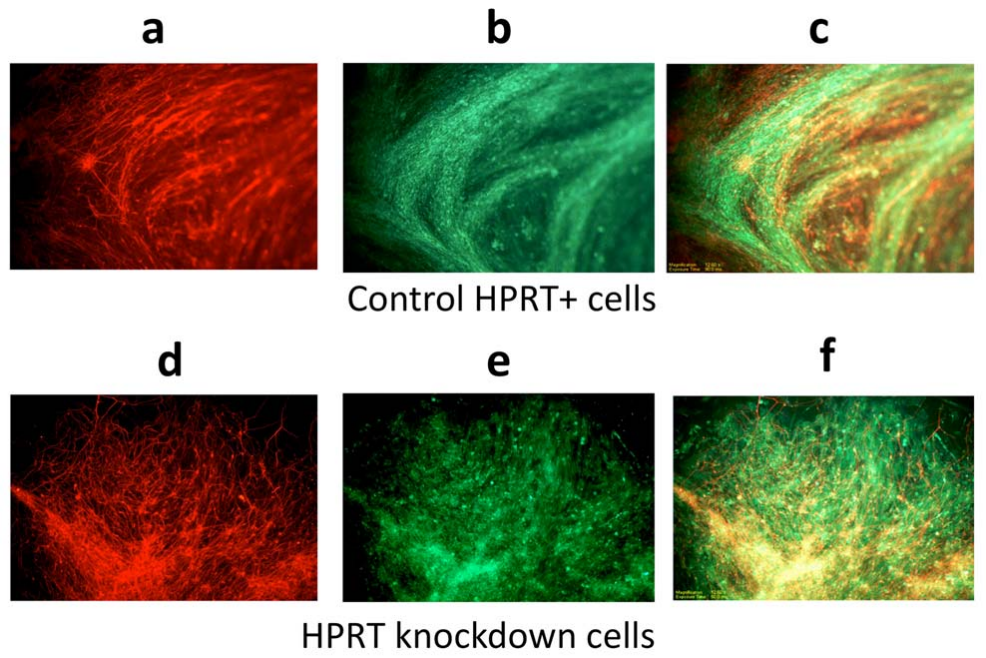
Immunohistochemical detection of the neuronal marker β-III tubulin and tyrosine hydroxylase (TH) in ESD3 cells at the final 14-day neuronal stage of SNM differentiation. (A) β-III tubulin in WT control cells; (B) TH expression in control WT cells; (C) merged β-III tubulin and TH expression in WT control cells; (D) β-III tubulin in HPRT-knockdown cells; (E) TH in HPRT-knockdown cells, and (F) merged β-III tubulin and TH expression in HPRT-knockdown cells.

**Figure 5:**
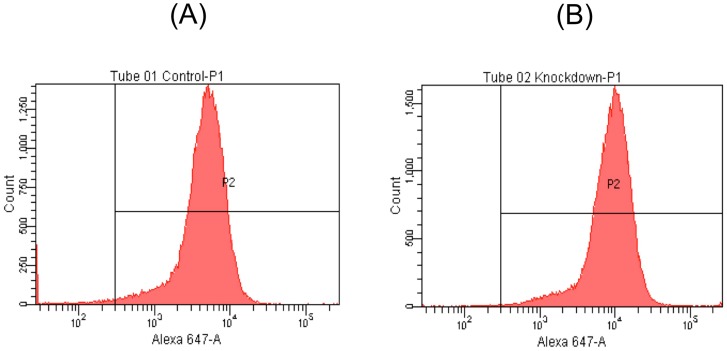
FACS analysis of control WT (A) and HPRT-knockdown (B) cells expressing TH. The cell types demonstrate indistinguishable high percentages of TH-positive cells.

To determine the efficiency of DA neurogenesis in WT and HPRT-knockdown cells, we used immunocytochemical methods to examine the fully differentiated neuron-like cells for expression of the DA neuronal markers β-III tubulin and the DA neuronal marker tyrosine hydroxylase (TH). Both markers were found to be readily expressed equally well in the WT and HPRT-knockdown cells at the final stage of differentiation (Fig. 4a, b, d, e). Merged images of TH-positive and β-III tubulin-positive cells demonstrated that a high percentage of cells derived from either the WT or the HPRT-knockdown cells co-express the two neuronal markers with approximately equal efficiency (Fig. 4c, f). Furthermore, FACS analysis with TH-specific antibody demonstrated that the efficiency of differentiation to the TH-positive phenotype was similar in the two cell types (∼97% and ∼99% of WT and HPRT knockdown cells, {Figure 5A and 5B, respectively}). The similarly efficient generation of TH and β-III tubulin-positive DA neuron-like cells from WT and HPRT-knockdown ESD3 cells is consistent with the fact that human LND brain and brains of HPRT-knockout mice display essentially normal cyto-architecture and contain largely normal numbers and normal distribution of TH-positive neurons [10]–[12]. These morphological studies do not test whether neurons generated from HPRT-deficient embryonic stem cells are functionally normal and display abnormal cellular migration or cell-interaction properties *in vivo* or are subject to the same normal cell death mechanisms during CNS development as cells derived from WT HPRT+ stem cells.

### Transcriptional aberrations

We characterized the transcriptomes of differentiating WT and knockdown cells by both microarray analysis and by RNA-Seq analysis. Microarray analysis of the parent WT and HPRT-knockdown cells was limited to the undifferentiated ES stage, the SNM stage and the final fully differentiated neuronal stage. To define the more detailed temporal gene regulatory changes induced by HPRT deficiency, we also performed RNA-Seq transcriptional analysis on days 0, 1, 2, 3, 4, 6, 8, 10, 12 and 14 of differentiation of SNM cells. Data derived from the microarray and the RNA-Seq experiments were analyzed by GeneSpring GX and Avadis NGS, respectively, and additional signaling pathway analyses were carried out using the Panther classification system.

The total number of genes differentially expressed at the 1.5X-fold level and their direction of change are summarized in Table 1. At the ES stage, 73 genes are dysregulated in knockdown ESD3 cells compared with WT cells, 13 of which are up- and 60 down-regulated. As the cells progress through the intermediate SNM stage of differentiation and then to the final neuronal stage, the knockdown cells demonstrate greater numbers of down-regulated (710) and up-regulated (1,318) genes at the SNM stage compared with the ES stage and 1,653 down-regulated and 1,485 up-regulated genes at the neuronal stage compared with WT cells.

**Table 1:** Number of genes found by microarray analysis to be differentially expressed in HPRT-knockdown ESD3 cells at the ES, SNM, and DA neuronal stages of differentiation.

Stage	Up	Down	Total
ES	13	60	73
SNM	1,318	710	2,028
DA	1,485	1,653	3,138

The up- and down-regulations are based on the HPRT knockdown condition.

We subjected the WT and HPRT-knockdown cell transcriptome databases to gene ontology (GO) analysis during the differentiation period from the embryonic stem cell stage to the DA neuronal stage. GO analysis of microarray data using GeneSpring GX revealed surprisingly that HPRT deficiency markedly perturbs a neuronal/glial cell fate decision that may have relevance to the neural defects in LND. Tables 2 and 3 demonstrate the evolution of expression of genes included in the Nervous System GO term during neuronal differentiation. Table 2 presents the GO terms that are differentially expressed (≥1.5 fold change) in SNM from WT and HPRT-knockdown ESD3 cells. At the SNM stage of differentiation, WT and HPRT-knockdown cells show virtually identical patterns of up-regulation of neural functions and CNS development GO terms. However, at the neuronal stage of differentiation, HPRT-knockdown cells demonstrate a striking down-regulation of virtually all of the terms related to neuronal functions in HPRT-knockdown cells compared with WT cells and a concomitant up-regulation of glial and myelination terms related to gliogenesis, myelination and ensheathment (Table 3).

**Table 2:** GO functions significantly altered (p≤0.05) in the GO term “nervous system” at the SNM stage of control and HPRT-knockdown cells.

*Up regulation in SNM stage in Control*		
*GO ACCESSION*	*GO Term*	*corrected p-value*
GO:0007399	nervous system development	6.51E-10
GO:0043005	neuron projection	2.18E-06
GO:0048699	generation of neurons	1.33E-05
GO:0022008	neurogenesis	2.16E-05
GO:0031175	neuron projection development	6.22E-05
GO:0048666	neuron development	1.11E-04
GO:0030182	neuron differentiation	1.12E-04
GO:0007409|GO:0007410	axonogenesis	0.00146468
GO:0048812	neuron projection morphogenesis	0.00408663
GO:0048667	cell morphogenesis involved in neuron differentiation	0.00532666
GO:0030424	axon	0.0127463
GO:0045202	synapse	0.02571509

*Down regulation in SNM stage in Control*		
*GO ACCESSION*	*GO Term*	*corrected p-value*
	No significant GO terms related nervous system	

*Up regulation in SNM stage in HPRT knockdown*		
*GO ACCESSION*	*GO Term*	*corrected p-value*
GO:0007399	nervous system development	1.97E-11
GO:0048699	generation of neurons	6.21E-06
GO:0022008	neurogenesis	7.68E-06
GO:0043005	neuron projection	4.99E-05
GO:0031175	neuron projection development	4.99E-05
GO:0048666	neuron development	1.38E-04
GO:0030182	neuron differentiation	1.38E-04
GO:0007409|GO:0007410	axonogenesis	0.00133804
GO:0048812	neuron projection morphogenesis	0.00154705
GO:0048667	cell morphogenesis involved in neuron differentiation	0.00238253
GO:0045202	synapse	0.00900807
GO:0030424	axon	0.046589

*Down regulation in SNM stage in HPRT knockdown*		
*GO ACCESSION*	*GO Term*	*corrected p-value*
	No significant GO terms related nervous system	

**Table 3:** GO functions significantly altered (p≤0.05) in the GO term “nervous system” at the DA neuronal stage of control and HPRT-knockdown conditions.

*Up regulation in DAstage in Control*		
*GO ACCESSION*	*GO Term*	*corrected p-value*
GO:0030665	clathrin coated vesicle membrane	4.77E-06
GO:0030135|GO:0005909	coated vesicle	7.19E-06
GO:0045202	synapse	9.55E-06
GO:0030136	clathrin-coated vesicle	9.65E-06
GO:0030662	coated vesicle membrane	4.24E-05
GO:0043005	neuron projection	0.001051372
GO:0044456	synapse part	0.001532143
GO:0030672	synaptic vesicle membrane	0.011576067
GO:0008021	synaptic vesicle	0.013373459

*Down regulation in DA stage in Control*	
*GO ACCESSION*	*GO Term*	*corrected p-value*
	No significant GO terms related nervous system	

*Up regulation in DA stage in HPRT knockdown*	
*GO ACCESSION*	*GO Term*	*corrected p-value*
GO:0008366|GO:0042553	axon ensheathment	0.006744339
GO:0007272	ensheathment of neurons	0.006744339
GO:0019228	regulation of action potential in neuron	0.011118365
GO:0043209	myelin sheath	0.04257185

*Down regulation in DA stage in HPRT knockdown*	
*GO ACCESSION*	*GO Term*	*corrected p-value*
GO:0007399	nervous system development	0.030906077
GO:0031175	neuron projection development	0.0315122
GO:0048812	neuron projection morphogenesis	0.036022328
GO:0007411|GO:0008040	axon guidance	0.046554424
GO:0007409|GO:0007410	axonogenesis	0.047252778
GO:0030182	neuron differentiation	0.0481869

**Figure 6:**
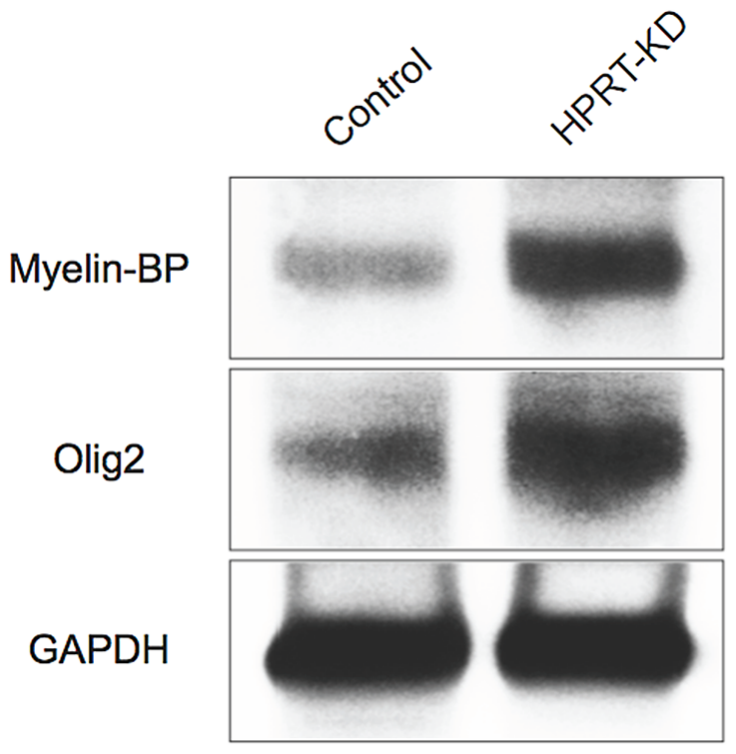
Western blot analysis for glial markers MBP and Olig2 of control WT cells and HPRT-knockdown ESD3 cells at the final neuronal stage of differentiation. Both markers demonstrate a significant increase of expression in the HPRT-knockdown cells.

For an even more detailed comparison of myelination-related functions in WT and HPRT-knockdown cells at the ES, SNM and neuronal stages, we carried out analyses using GeneSpring GX as presented in Table 4. This analysis identifies no differential gene expression at the ES stage in either WT or HPRT-knockdown ESD3 cells but confirms altered gene expression at the SNM and neuronal stages of differentiation of a number of myelination-related pathways such as those associated with Notch, hedgehog and histone deacetylase (HDAC) pathways. Western blot analysis of lysates of WT and HPRT-knockdown at the neuronal stage of differentiation provides support for a functional consequence of the up-regulated glia-related gene expression in the HPRT knockdown cells by demonstrating increased levels of the two key myelination functions, MBP and Olig2 in the HPRT-knockdown cells (Fig. 6).

**Table 4:** Significantly altered (p≤0.05) myelination-related pathways in HPRT knockdown cells.

*In embryonic stage*
*Pathway*	*corrected P-value*
No significant pathway related to myelinating related pathways	

*In SNM stage*
*Pathway*	*corrected P-value*
Maturation of Notch precursor via proteolytic cleavage	0.015534699
Notch signaling pathway	0.017844982
Signaling events mediated by the Hedgehog family	0.018737104
Signaling events mediated by HDAC Class II	0.025368122

*In DA stage*
*Pathway*	*corrected P-value*
Sonic hedgehog receptor ptc1 regulates cell cycle	0.011203819
Signaling events mediated by HDAC Class III	0.04690445

To test the possible relevance of these aberrations to DA neurotransmission functions, GO analyses of the transcriptomes from microarray data were performed using Avadis NGS. These analyses reveal up-regulated expression of multiple DA and catecholaminergic functions at the neuronal stage of differentiation of WT cells but reduced up-regulation in HPRT knockdown cells (Table 5).

**Table 5:** Expression of “dopamine” GO terms in differentiated neuronal cells.

*Up-regulation in SNM day 14 in Control*	
*GO Term*	*corrected p-value*
synaptic transmission, dopaminergic	3.11E-04
dopamine binding	8.35E-04
dopamine receptor activity	0.00103916
catechol metabolic process	0.01598897
catecholamine metabolic process	0.01598897

*Up-regulation in SNM day 14 in HPRT knockdown*	
*GO Term*	*corrected p-value*
dopamine biosynthetic process	0.00705662

WT control cells demonstrate up-regulation of multiple dopamine and catecholinergic functions while HPRT-knockdown neuronal cells demonstrate a narrower pattern of up-regulated dopamine biosynthetic processes. Neither cell type demonstrates down-regulation of dopamine GO terms.

A more detailed extension of these findings to additional neurotransmitter pathways is demonstrated by GO analysis of RNASeq data and demonstrates that the differentiated HPRT-knockdown neuronal cells demonstrate aberrant neurotransmitter GO terms related to glutamatergic, serotonergic and GABAergic functions (Tables S2–4 in File S1 respectively). The pattern of dysregulation in HPRT-deficiency is similar for all three neurotransmitters; i.e., very little change in WT cells until late in the differentiation process but marked and less apparently organized dysregulation throughout most of the differentiation process in HPRT-knockdown cells. In contrast to glutamatergic, serotonergic and GABAergic neurotransmitters, GO terms related to acetylcholinergic neuronal functions showed no consistent changes in either cell type.

Because it is generally thought that the underlying defect responsible for LND ultimately reflects aberrant purine metabolism, especially of guanine-based purines, and because of the possibility that altered GTP/GDP fluxes might therefore play a role in HPRT neuropathology [13], we examined the microarray data of WT and HPRT knockdown cells for the effect of HPRT knockdown on G-protein coupled receptor (GPCR) GO terms. While cells at the ES and intermediate differentiation SNM stages showed few differentially expressed GPCR-related functions, the late DA neuronal stage of differentiation revealed a large number of dysregulated GPCR-related functions (Table 6) and mapping of these functions to the Kyoto Encyclopedia of Genes and Genomes (KEGG) signaling pathway maps confirmed dysregulation of multiple calcium-mediated GPCR functions and signaling pathways in HPRT deficient cells.

**Table 6:** Significantly altered (p≤0.05) GPCR signaling pathways in control and HPRT-knockdown cells.

*In embryonic stage*	
*Pathway*	*corrected P-value*
No significant pathway related to GPCR	

*In SNM stage*	
*Pathway*	*corrected P-value*
Wnt signaling pathway	9.45E-03
Heterotrimeric G-protein signaling pathway-Gi alpha and Gs alpha mediated pathway	1.67E-02

*In DA stage*	
*Pathway*	*corrected P-value*
Metabotropic glutamate receptor group III pathway	1.98E-06
Wnt signaling pathway	2.77E-06
5HT2 type receptor mediated signaling pathway	6.94E-06
Thyrotropin-releasing hormone receptor signaling pathway	7.74E-06
Cytoskeletal regulation by Rho GTPase	3.54E-05
Histamine H1 receptor mediated signaling pathway	3.62E-05
Oxytocin receptor mediated signaling pathway	5.20E-05
Beta1 adrenergic receptor signaling pathway	5.88E-05
Beta2 adrenergic receptor signaling pathway	5.88E-05
5HT1 type receptor mediated signaling pathway	1.78E-04
GABA-B_receptor_II_signaling	2.38E-04
Metabotropic glutamate receptor group II pathway	6.31E-04
Heterotrimeric G-protein signaling pathway-Gi alpha and Gs alpha mediated pathway	7.36E-04
Ras Pathway	9.44E-04
Endothelin signaling pathway	1.09E-03
Heterotrimeric G-protein signaling pathway-Gq alpha and Go alpha mediated pathway	1.12E-03
5HT4 type receptor mediated signaling pathway	1.18E-03
Beta3 adrenergic receptor signaling pathway	1.60E-03
Histamine H2 receptor mediated signaling pathway	2.15E-03
Heterotrimeric G-protein signaling pathway-rod outer segment phototransduction	6.94E-03
Metabotropic glutamate receptor group I pathway	7.96E-03

Because we have previously reported that HPRT deficiency leads to several aberrant signaling defects in common with those found in Alzheimer's disease, as in the case of dysregulated presenilin-1 and Wnt/β-catenin signaling in HPRT-deficient fibroblasts [6], we applied the Panther Classification System to examine the microarray transcriptome data for GO terms related to neurodegenerative disease (Table 7). Most striking in this analysis are the findings at the embryonic stem cell and neuronal differentiation stages of HPRT-deficient cells of dysregulated signaling pathways also aberrantly expressed in Alzheimer's and Huntington's disease, including the presenilin-1 pathway, lending support to a possible functional significance of our previous findings in HPRT-deficient fibroblasts [6] and supporting the concept that LND, Alzheimer's and Huntington's diseases may share some common pathogenic mechanisms.

**Table 7:** Significantly altered (p≤0.05) signaling pathways related to GO term “neurodegenerative disease” in control and HPRT-knockdown cells.

*In embryonic stage*	
*Pathway*	*corrected P-value*
Alzheimer disease-presenilin pathway	1.66E-03
Huntington disease	6.23E-03
Plasminogen activating cascade	3.31E-02

*In SNM stage*	
*Pathway*	*corrected P-value*
No significant pathway related to neurodegenerative disease	

*In DA stage*	
*Pathway*	*corrected P-value*
Alzheimer disease-presenilin pathway	1.39E-05
Alzheimer disease-amyloid secretase pathway	3.79E-05
Muscarinic acetylcholine receptor 1 and 3 signaling pathway	6.20E-05
Huntington disease	2.28E-04
Muscarinic acetylcholine receptor 2 and 4 signaling pathway	4.40E-04

**Figure 7:**
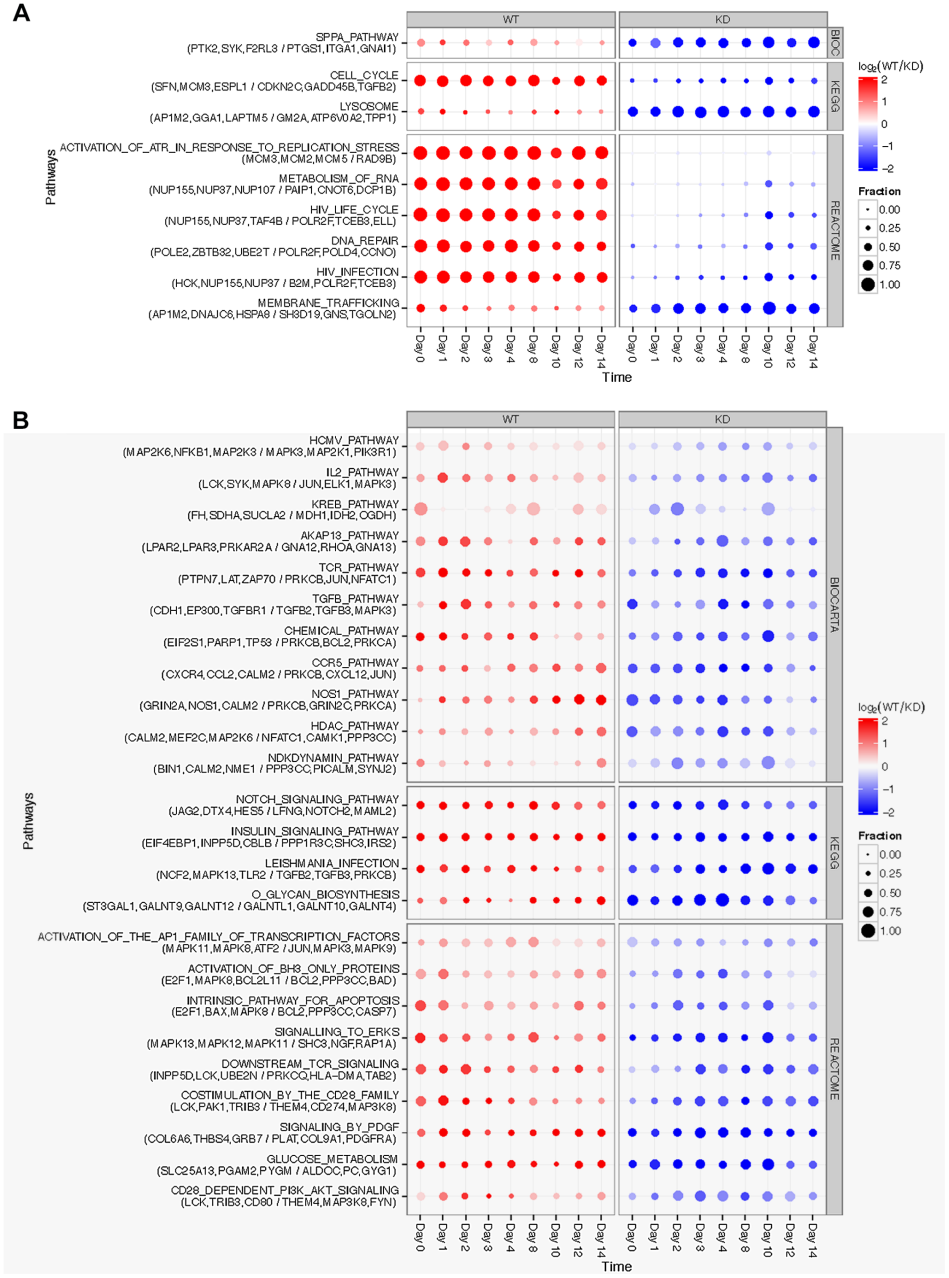
Mean shift and polarization of canonical pathways. Gene expression of 802 canonical pathways was tested for (A) shift in mean expression and (B) polarization, each test aggregating paired differences between wild-type (WT) and HPRT-knockdown (KD) at each time point during the differentiation. Rows are significant pathways, organized by source database and with the top three WT and KD genes provided underneath the pathway name. Columns are the module response over time, with red/blue indicating over-expression in WT/KD cells and diameter indicating the fraction of genes in the pathway with differential expression. The mean-shift test statistic is the mean log-ratio of WT to KD expression aggregated over time points for genes within a pathway; the polarization test statistic is the variance of gene log-ratios within a pathway. For both tests, the null distribution is obtained by permutation of sample labels. Thresholds are two false discoveries (FDR  =  0.22) for mean shift and one false discovery (FDR  =  0.042) for polarization.

To further define the unexpectedly broad and important regulatory role that HPRT seems to play in many cellular developmental and signaling processes, we examined dysregulation of canonical pathways from MSigDB [14] testing the RNASeq transcriptomic data for overall shift in mean and for polarization [15], and used sample-level permutations to establish stringent significance thresholds. Results are presented in Figure 7. At a threshold of two false discoveries, equivalent to a false-discovery rate of 0.22, we identified 6 major pathways that show consistent and temporally-related up-regulation in WT cells and 3 in KD cells. The 6 up-regulated WT pathways all reflect processes characteristic of rapidly proliferating cells: cell cycle, RNA metabolism, DNA repair, replication stress, and HIV life cycle and HIV infection pathways. The HIV-related pathways are not related to the immune response but rather to active transcription and translation, including over-expression of nuclear pore proteins, and also over-expression of the HCK gene, an Src protein-tyrosine kinase that activates microglial cells [16] and signals through PI3K/Akt [17]. The WT pathways are most active through day 8, decrease thereafter and then show a second increase at day 12 (Fig. 7).

The three pathways showing major up-regulation in KD cells (Fig. 7) include those characteristic of mechanisms related to lysosome function, membrane trafficking, and signaling pathway for platelet activation (SPPA). The most highly expressed lysosome genes are related to lysosomal function in sphingolipid and ganglioside metabolism that are active in astroglial cells [18]. Similarly, the most highly expressed membrane trafficking genes such as GNS are related to sulfate metabolism in the lysosome and storage disorders.

The polarization test identifies pathways with subsets responding in opposite directions, thereby resulting possibly in no net shift in overall pathway expression. Polarization often arises in cell signaling, with distinct cell types expressing ligands and receptors, and systematic differences are apparent. Detailed analysis of results illustrated in Fig. 7 have identified a number of such polarized gene expression aberrations (data not presented). WT cells express Notch ligands jagged-2 (JAG2) and deltex-4 (DTX4) whereas HPRT-KD cells express notch 2, notch glycosylation protein Lunatic Fringe (LNFG), and notch signaling partner mastermind-like protein 2 (MAML2). HPRT-KD cells express CXCL12, whose receptor CXCR4 is highly over-expressed in WT cells. HPRT-KD cells also express TGFB2 and TGFB3, whose receptor TGFBR1 is over-expressed in WT cells. Polarization may also reflect pathways that include both activators and deactivators/repressors. For example, in the T cell receptor (TCR) signaling pathway, WT cells express protein tyrosine phosphatase non-receptor type 7 (PTPN7), which negatively regulates MAPK1/ERK2 and MAPK3/ERK1 [19], [20] (Table 7), whereas HPRT-KD cells over-express MAPK3. These transcriptional findings support and suggest possible mechanisms responsible for our previous Western blotting demonstration of constitutive over-expression of MAPK/ERK1 and ERK2 in HPRT-deficient iPS cells [7]. Polarization also arises from expression of alternative subunits. For example, the NOS1 pathway points to HPRT-regulated discordant expression of NMDA glutamate receptors: WT cells express increasing amounts of GRIN2A, whereas HPRT-KD cells express GRIN2C and GRIN3A (Fig. 7).

## Discussion

Studies of the classical monogenic HPRT-deficiency Lesch Nyhan Disease have largely failed previously to connect the well-understood underlying genetic defect of purine salvage biosynthesis with the development of the neurological defects of dystonia, choreoathetosis, moderate cognitive impairment and compulsive self-mutilation. We have recently identified a number of biochemical and molecular aberrations in HPRT deficiency that point to multiple defects in gene expression and cellular signaling involving expression of DA transcription factors, a variety of signaling pathways and miRNA expression. We have hypothesized that these defects may contribute to severe developmental and functional dysregulation of neural developmental pathways, especially those that determine DA neurogenesis and dopaminergic signaling. We have now used a robust system for *in vitro* neuronal differentiation of iPS and ES cells to identify some mechanisms by which HPRT regulates the molecular and cellular functions during neuronal differentiation of pluripotent murine ESD3 cells. These results establish that the housekeeping gene HPRT carries out functions far more complex than simply driving the metabolic steps of salvage purine biosynthesis and that it also plays very important roles in regulating a large number of developmental and cell signaling pathways that accompany and regulate many aspects of CNS development, most particularly mechanisms that determine neuronal/glial cell fate decisions during neurogenesis. We have used global transcriptomic characterization methods to identify mechanisms that are severely disturbed when HPRT expression is deficient. Although we have not yet fully established a direct causal relationship between these aberrations and the development of the neural dysfunction in the HPRT-deficient mammalian CNS, we have built upon these findings in the murine HPRT deficiency model to develop a working hypothesis that important aspects of impaired development and function of DA and possibly other neural pathways in human HPRT deficiency result from aberrations of many pathways and signaling systems caused by dysregulated differentiation and cell fate decisions made by embryonic stem cells during neurogenesis in the developing embryonic human CNS.

One major prime candidate for playing a causative factor in defective neural development in HPRT deficiency is an apparently aberrant embryonic cell fate decision that leads to a switch from a neuronal pattern of gene expression to an almost exclusively glial pattern of gene expression. Approximately midway during the ES DA neuronal differentiation process in vitro, HPRT-deficient murine ES cells undergo a major transcriptional switch away from neuronal almost entirely to a glial gene expression program, even as they continue to generate cells with at least some of the principal molecular properties of dopaminergic neurons such as β-III tubulin and TH expression (Fig. 4). We note the apparently discrepant results of β-III tubulin expression as determined by qPCR (Fig. 3) and immunostaining (Fig. 4) techniques. We do not have experimental results that explain that difference, but several previous reports have demonstrated that such discrepancies between mRNA and protein gene product levels are well recognized in some systems [21], [22]. The exact nature of the responsible mechanisms has not been definitively established in these earlier studies, but the differences have been attributed to many possible mechanisms, including actual differences between the transcripts and protein products reflecting the existence of possible alternative differential splicing events leading to multiple isoforms with different mechanisms regulating their protein stability and abundance, probe set definition, technological sensitivity differences, etc.

Although the switch from neuronal to glial gene expression might be expected to affect the generation or distribution of glial cells and altered myelination and ensheathment functions in HPRT deficiency, there have been until now no major neuronal or glial cyto-architectural aberrations described in HPRT-knockout mice and only occasional but unconfirmed demonstrations of glial or myelination disturbances with peripheral neuropathy in human LND patients [23], [24]. Multiple mechanisms may theoretically cooperate to dysregulate gliogenesis and myelination in HPRT deficiency. Wnt signaling plays a vital role in myelination in the mammalian CNS [25]–[32] and the Wnt/β-catenin canonical signaling pathway inhibits oligodendrocyte maturation and their myelination function [30], underscoring the possible significance of our previous report of deficient canonical Wnt-1 signaling/β-catenin processing in HPRT-deficiency [6]. This impaired β-catenin expression may therefore play a role in up-regulating glial gene expression in the HPRT-knockdown ESD3 cells described above. In addition, the dysregulated myelination-associated signaling pathways shown in Table 4 include aberrations of histone deacetylase (HDAC), hedgehog and Notch function, all of which are known to be regulators of myelination and glial function.

Mice lacking both HDAC1 and HDAC2 exhibit severe myelin deficiency with arrested Schwann cell development and HDAC2 acts in synergy with Sox10 to activate the transcriptional program of myelination [30], [33], [34]. Since hedgehog and Notch participate in the regulation of oligodendrocyte function and myelination, their perturbations in HPRT deficiency could contribute to mechanisms producing the neuronal/glial gene expression switch described in the present murine ES cell model system. Hedgehog expression is required for the expression of Olig1 and Olig2 in myelin-forming oligodendrocytes [35]–[38] and the aberrant hedgehog expression seen in the HPRT-knockdown ESD3 cells might therefore be expected to alter oligodendrocyte functions and mechanisms of myelination. Similarly, Notch expression is required by oligodendrocytes for production and stabilization of the myelin component myelin basic protein (MBP) [39]. The increased level of MBP in the HPRT-knockdown cells illustrated in Fig. 6 may therefore be at least partly the result of dysregulated Notch expression (Table 4) and subsequent to up-regulated expression of MBP. Interestingly, despite the robust aberration of gliogenic gene expression in the differentiating murine ES cells, no major myelination and gliogenesis defects have been described in HPRT deficiency, either in the human or in the HPRT-knockout mouse. Only two studies have reported possible myelination aberrations in HPRT deficiency [23], [24] but the question of gliogenesis and myelination have not been thoroughly examined in HPRT deficiency. Nevertheless, our present studies demonstrate that HPRT is a strong determinant of the neuronal or glial gene expression pattern in murine ES cells during neuronal differentiation. That fact together with dysregulations in multiple other regulatory systems such as notch, hedgehog and HDAC, in HPRT deficiency (Table 4), indicates the need for future studies to identify the specific aberrations in these signaling pathways and to determine whether they cause, follow or are merely coincidentally related to the altered patterns of gene expression in HPRT-deficient cells, both in cell culture model systems and in vivo.

Another surprising defect in differentiating HPRT-deficient murine ES cells is the aberrant regulation of Sox2. The aberrant neuronal/glial developmental switch may be mechanistically related to an altered state of pluripotency and aberrant mechanisms of exit from pluripotency and initiation of neuronal differentiation in HPRT-deficient ESD3 cells, as suggested by the early and progressive up-regulation of Sox2 during neuronal differentiation (Fig. 3C). Sox2, along with Nanog and Oct-3/4, are vital for faithful mammalian embryogenesis, for the maintenance of pluripotency and for aspects of neural development [40]–[44]. Persistent expression of Sox2 is associated with continued proliferative potential of stem cells while down-regulation is characteristic of cells differentiating into post-mitotic neurons. Equally striking is the demonstration that Sox2 over-expression interferes with neurogenesis in both the mouse and the chicken inner-ear [45], [46] and that production of neurons from stem cells requires down-regulation of Sox2. Therefore, the persistent and even increasing expression of Sox2 in the differentiating HPRT-deficient ESD3 cells in these studies is consistent with the impaired neurogenesis characteristic of HPRT deficiency [2]–[5].

The extreme complexity of this system is underscored by the apparent discrepancy between what seems to comparably efficient generation of differentiated cells with morphological and molecular and properties of DA neurons (Figs. 2,4 and 5) and the sharp shift during DA neurogenic differentiation away from a neuronal to a glial pattern of gene expression. While these and our previous studies have identified a degree of aberrant DA neurogenesis in HPRT deficiency, the defect is an impeded, misdirected or inappropriately timed differentiation rather than a block to neurogenesis. The CNS in LND and in HPRT-knockout mice are known to contain normal numbers and distribution of cells with morphological properties of dopaminergic neurons and our own studies show generation from HPRT-deficient murine ES cells of populations of cells which express the DA neuronal markers TH and β-III tubulin cells but which have virtually shut down neuronal gene expression in favor of glial cell gene expression. This apparent discrepancy in HPRT-regulation of DA neurogenesis may be explained partly by the possibility that most cells in a heterogeneous population of differentiated HPRT-deficient DA-neuronal cells have become partially differentiated toward the DA neuronal phenotype, as least far enough along the pathway to express β-III tubulin TH and but are being impeded by HPRT deficiency and are either being re-directed along a glial developmental pathway or are being replaced by glial elements in the cell population that have a growth or survival advantage in the HPRT-deficient milieu.

An additional intriguing clue to the mechanisms of neuropathogenicity in HPRT deficiency is provided by the broad dysregulatory effect of HPRT deficiency on guanine purine metabolism and thereby on GPCR expression. HPRT deficiency causes broad changes in purine and purine nucleotide levels, including guanine nucleotide levels [47], an effect that would be expected to dysregulate the function of many GPCRs and thereby to disturb many cellular signaling transduction events dependent on intact GPCR signaling pathways. We have previously reported that HPRT deficiency leads to defective expression of the GPCR purinergic receptor P2Y1 and that in turn is associated with aberrant pERK and pCREB signaling [7]. Other groups have shown that HPRT alters activities of additional GPCRs including membrane NTPase and adenylate cyclase [48], [49] The many aberrantly-expressed GPCRs listed in Table 7 include functions associated with neurotransmission and neural development, including Wnt-1 signaling, glutamate receptors, 5HT receptors, GABAergic receptors. These results are consistent with our findings of disturbed function of these neurotransmitter functions as identified in Tables S2–4 in File S1, with aberrant regulation of aspects of DA binding, DA receptor activity, and catechol metabolic processes. Our findings also support previous demonstrations by other groups of defective function of serotonin and adenosine receptors in HPRT deficiency [50]–[53]. Overall, these studies indicate that HPRT deficiency produces neurotransmission defects more extensive than the well-recognized defects in dopamine transmission and DA neurogenesis.

As illustrated in Fig. 7, HPRT deficiency in murine ESD3 cells is associated with impaired cellular activity devoted to an unexpectedly broad set of signaling pathways and metabolic functions that affect cell proliferation, protein synthesis, RNA biosynthesis, DNA replication and repair, enhanced cellular effort devoted to such diverse functions as sphingolipid metabolism and membrane biosynthesis, sulfur and glycan metabolism and to PPAR metabolism [54], [55] and others. In several of the cell signaling pathways, the wild-type and HPRT-deficient cells express a number of discordant component mechanisms potentially participating in aberrant neuronal development. These systems have not been thoroughly characterized in human HPRT deficiency and their functions and interactions in producing the HPRT neural phenotype are largely unknown.

An area of potential relevance to HPRT neuropathology is the possibility that HPRT deficiency leads to disruption of normal microRNA expression, thereby leading to aberrant expression of many target genes involved in embryonic development of the mammalian CNS. Defects in miRNA expression would have the effect of providing an efficient mechanism for producing the extremely broad set of downstream defects that characterize HPRT deficiency and the LND neurological phenotype. We have previously reported that HPRT deficiency indeed leads to dysregulation of at least one miRNA (miR181a) [8] and recently we have found preliminary evidence for dysregulated expression of additional miRNAs (unpublished results). Their significance is under study.

We interpret these combined results to indicate that the housekeeping gene HPRT is a vital neurodevelopmental gene and that it plays a number of important non-“housekeeping” functions. Our studies suggests that HPRT is a determinant in regulating steps in mammalian CNS development possible through combined effects on stem cell pluripotency and neuronal differentiation, on cell fate decisions in neuronal and glial development, on microRNA expression, on GPCR expression, and on multiple additional cellular signaling and metabolic functions. We surmise that HPRT deficiency constitutes a systems disorder and that the pathogenesis of this monogenic but yet very complex neurodevelopmental Mendelian disease results from combinatorial and multigenic defects. We further surmise that these defects are all ultimately the result of aberrant purine metabolism but we cannot exclude the possibility that at least some of the cellular defects may result from other pleiotropic and non-purinergic functions of the HPRT protein. Nevertheless, to our knowledge, this is the first time this kind of stem cell developmental gene expression analysis has been carried out in a neurodevelopmental disease and certainly in HPRT deficiency, and we suggest that it is of great value to workers in this field that we have documented the broad transcriptional aberrations induced in many pathways by HPRT deficiency. Our study identifies, for the first time, the many pathways that now can become accessible for deeper functional study and underscores the importance of examining functions in all of these aberrant pathways if we are to understand the very complex mechanisms by which the purine metabolome regulates neurogenesis and its role in the pathogenesis in Lesch Nyhan Disease.

## Materials and Methods

### Cells and neuronal differentiation

The ESD3 mouse embryonic stem cell (mESC) line derived from mouse strain 129s2/SvPas was purchased from ATCC (Manassas, VA) and the cells were maintained on primary mouse embryo fibroblast (PMEF) feeder cells in ES medium consisting of Dulbecco's minimum essential medium (DMEM)/F12 medium, 20% (v/v) knockout serum replacement (KOSR), 1 mM L-glutamine, 4 ng/ml of basic fibroblast growth factor (bFGF), 1,000U/ml of leukemia inhibitory factor (LIF), 1% (v/v) nonessential amino acids (NEAA), 50 U/ml of penicillin, 50ug/ml of streptomycin and 0.1 mM of 2-mercaptoethanl. PMEF and LIF were obtained from Millipore (Billerica, MA), and the other reagents used for ES medium were obtained from GIBCO (Grand Island, NY). The culture and differentiation reagents including collagenase IV, N2 supplement and B27 supplement were purchased from GIBCO and sonic hedgehog (Shh), fibroblast growth factor 8 (FGF8) and ascorbic acid were obtained from R&D system (Minneapolis, MN, USA), PeproTech (Rocky Hill, NJ) and Sigma (St. Louis), respectively. Matrigel was purchased from BD Biosciences (San Jose). All cell culture and neuronal differentiations were carried out in established cell culture conditions (5% CO_2_, 37°C). Dopaminergic (DA) neuronal differentiation was carried out according to the protocol published that allows highly efficient differentiation of DA neurons from embryonic bodies by expansion of spherical neural masses (SNMs) [9].

### Preparation of control and HPRT-knockdown cells

The ESD3 cells were transduced with VSG-G-pseudotyped lentiviral vectors expressing shRNAs targeted against either luciferase or human HPRT genes, as reported [6], [7] and produced by the UCSD Gene Therapy Program Vector Core Laboratory. Transduced cells were selected with puromycin and assayed for HPRT as previously described (6). An additional possible control would include HPRT-knockdown cells in which HPRT activity is restored by transduction with an HPRT-expressing transgene. Interpretation of studies in such cells would potentially be complicated by the fact the HPRT knockdown in D3 cells has been achieved by expression of a retrovirally-delivered shRNA against HPRT which would continue to be expressed in HPRT-reconstituted cells and would therefore be available for shutting down HPRT transgene expression.

### Microarray analysis

Total RNAs from both HPRT knockdown and control cells were collected at the embryonic, SNM and final neuronal differentiation stages using RNeasy Mini Kit from QIAGEN (Valencia, CA). Purified RNAs from each condition were pooled and transferred to the UCSD Biogem Illumina core facility for gene expression analysis. The integrity of RNA samples was documented by Agilent Bioanalyzer (Santa Clara) and RNA preparations with RNA integrity numbers (RIN) of at least 9.2±0.5 were used for microarray analysis. Transcriptional analysis was performed in triplicate using the Illumina MouseWG-6 v2.0 Expression BeadChip Kit (≥ 45,200 transcripts) and reagents and experimental methods according to manufacturer's directions (Illumina, San Diego). Scanning of hybridized BeadChip and image quantitation was performed using Illumina's BeadArray software, raw data were normalized and exported to GeneSpring GX 11.5 (Agilent, Santa Clara) and the PANTHER Classification System for GO, GSEA and signaling pathway analysis (http://www.pantherdb.org/). Total detected entities were filtered by signal intensity value (upper cut-off 100^th^ and lower cut-off 20^th^ percentile) and error (coefficient of variation: CV <50.0 percent) to remove very low signal entities and to select reproducible signal values of entities among the replicated experiments, respectively. For statistical analysis, paired and unpaired t-tests (p<0.05) were used and significant changes were defined as above either 1.5-fold or 2-fold change. Signals were selected if they were above microarray background (detection p-value <0.05) in either all experiments or in at least three knockdown or control experiments in each pair for comparison. All microarray-based transcriptomic data are accessible through GEO Series accession number (GSE42453), a MIAME compliant database (e.g. ArrayExpress, GEO), as described on the MGED Society website (http://mged.sourceforge.net/ontologies/).

### RNA-Seq analysis

For RNA-Seq gene expression analysis of cells during differentiation from SNM to the final neuronal differentiation stage, total RNAs were collected as for microarray analysis during SNM differentiation at days 0, 1, 2, 3, 4, 6, 8, 10, 12 and 14. Purified RNAs derived from each time point in each condition were pooled and applied for RNA sequencing in the UCSD Biogem Illumina core facility. Library preparation was performed with TruSeq RNA Sample Preparation Kit v2 of Illumina (San Diego), and the prepared libraries were validated and quantified with Agilent Bioanalyzer (Santa Clara). The RIN was 9.5±0.5. To quantify and qualify the DNA library before sequencing, quantitative PCR and Nanodrop/Bioanalyzer analyses were carried out and the validated library was applied to the HiSeq 2000 sequencer of Illumina for 50 cycles of single lane run. Twenty libraries were applied to four sequencing lanes evenly in a flow cell and five samples in a lane were distinguished by using index libraries. Raw sequencing data (fastq.gz file) were generated by CASAVA 1.8.2 pipeline provided by Illumina and aligned onto NCBI37/mm9 mouse genome using ELAND (Illumina) alignment algorism in the pipeline (txt.gz file).

Aligned data including all aligned reads, the partial reads, novel gene and exons were imported to Avadis NGS 1.3.1 (Strand Scientific Intelligence, San Francisco). An average of 15 million reads by Illumina tile plot filter were applied to following analysis. The parameters used for finding novel genes and exons included one 10^th^ of minimum exon, intron and gene lengths, one 50^th^ of minimum exon and gene RPKM (reads aligned per kilobases per million mapped), one 75^th^ of minimum exon RPKM w.r.t. host gene and one 90^th^ of maximum intron length, were specified. The normalization algorithm was DESeq (http://www.bioconductor.org/). To remove very low signal entities, total detected entities were filtered by gene expression values (upper cut-off 100^th^ and lower cut-off 20^th^ percentile) and required at least one out of two samples to have values within that range. To detect differential gene expression during neuronal differentiation, changes either ≥1.5 or ≥2.0 fold between control and HPRT knockdown conditions were selected. Analyses of GO, GSEA and signaling pathways were carried out using the Avadis NGS 1.3.1 (Strand Scientific Intelligence) and the PANTHER Classification System (http://www.pantherdb.org/). All transcriptomic data are MIAME compliant and raw data were deposited in NCBI's Gene Expression Omnibus [54] and are accessible through GEO Series accession number (GSE42662), a MIAME compliant database (e.g. ArrayExpress, GEO), as detailed on the MGED Society website (http://mged.sourceforge.net/ontologies/).

Canonical pathways from MSigDB [14] were also analyzed. For this analysis, over 98% of short reads were mapped to NCBI mm9 transcripts using Tophat [55]; gene-level RPKM values were provided by Cufflinks [56]; and gene-level differential expression log-ratios, z-scores, and p-values for knockdown vs. control at each time point were estimated by Cuffdiffs. The day 6 data appeared to be a highly discrepant outlier and were omitted. Pathways were required to have at least 10 observed genes, yielding 802 for testing. For each pathway, two tests were performed: shift-of-mean, for overall up- or down-regulation of the entire pathway, and polarization, for subsets of genes with strong up-regulation and down-regulation within a single pathway. Both tests used parametric statistics [15] and corrected for gene non-independence by permutation.

The test statistic for shift-of-means was *S*  =  S*_gt_z_gt_*/(*GT*)^1/2^, where *z_gt_*is the z-score for wild-type (WT) vs. knockdown (KD) for gene *g* at time *t*, and the sum is over the *G* genes in a pathway and *T* total time points, and using if the z-scores are independent and identically distributed standard normal, the normalization by (*GT*)^1/2^ yields a standard normal. To correct for non-independence, however, we performed all 2^9^ sample-level permutations by swapping WT and KD labels within time points, corresponding to negating all z-scores at that time point, but excluded the permutations equivalent to the observed data and to the negation of the observed data. The mean m and variance s^2^ of *S* were computed for each pathway individually from the 510 permutations; *S* for each pathway was converted to a z-score (*S*–m)/s; and a two-sided p-value for *S*≠mwas calculated from the standard normal distribution. The p-value for one false discovery was estimated as 1/(number of pathways tested), or 1/802. No pathway was significant at this level, but 9 pathways were significant at two false discoveries, equivalent to a false-discovery rate of 0.22.

The test statistic for polarization was *P*  =  [S*_g_* (*z_g_*–z)^2^– (*G*–1)]/[2(*G–*1)], where the z-score for a gene is *z_g_*  =  S*_t_z_gt_*/*T* ^1/2^, and the mean dysregulation for a pathway is z  =  S*_g_z_g_*/*G*. If the *z_gt_* values are independent, identically distributed standard normal, then S*_g_* (*z_g_*–z)^2^ follows a chi-square distribution with *G*–1 degrees of freedom; subtracting the mean *G*–1 and dividing by variance 2(*G*–1) gives a test statistic that approaches a standard normal. To correct for non-independence, the mean m and variance s^2^ of *P* were computed for each pathway with permutations; *P* was converted to a z-score as (*P*–m)/s; and the one-sided p-value for *P*>m was calculated from the standard normal distribution. At one false discovery, 24 pathways were significant, equivalent to a false-discovery rate of 0.042.

### Immunocytochemistry

Cells maintained on Matrigel-coated coverslips (BD Biosciences, San Jose, California, USA) were stained and examined as described [15]. Mouse monoclonal anti-beta III tubulin and rabbit monoclonal anti-tyrosine hydroxylase primary antibodies in this study were purchased from Abcam (Cambridge), and the anti-mouse and anti-rabbit IgGs conjugated to Texas Red and FITC, respectively, were obtained from Santa Cruz Biotechnology (Santa Cruz). Expression of DAPI and secondary fluorophores was detected using aBX51 fluorescence microscope digital imaging system of Olympus (Center Valley, PA).

### FACS analysis

For FACS analysis, approximately 10^6^ cells were fixed by 4% paraformaldehyde (Sigma) for 15 minutes at room temperature, permeabilized with 100% methanol on ice for 30 min. Treated cell aliquots were exposed to blocking solution of 0.5% BSA (Sigma) in PBS for 10 min at room temperature. For immunostaining, primary antibodies were diluted in blocking solution according to the manufacturer's instructions and incubated with cells for 1 hour at room temperature. The fluorochrome-conjugated secondary antibody was applied for 30 min at room temperature. Antibodies included rabbit monoclonal anti-tyrosine hydroxylase primary antibody (Abcam) and Alexa Fluor 647-conjugated anti-rabbit IgG (Life Technologies, Grand Island, NY). Analysis of immunostained cells was carried out in the flow cytometry core facility in the UCSD Moores Cancer Center using FACSCalibur flow cytometer of BD Bioscience (San Jose).

### Quantitative PCR analysis

Purification of total RNAs in cell extracts was carried out by RNeasy Mini Kit of QIAGEN (Valencia) according to the manufacturer's instruction and qPCR reactions were performed using glyceraldehyde-3-phosphate dehydrogenase as a standardization control. Primer sequences are summarized in Table S1 in File S1.

### Western blotting

Western blotting studies were carried out as previously described [6]. The rabbit polyclonal HPRT, and antibodies to myelin basic protein and olig2 were purchased from Abcam and the loading control, mouse monoclonal GAPDH antibody was obtained from Cell Signaling Technology (Danvers, MA). The rabbit and mouse IgG-HRP secondary antibodies were purchased from Thermo Scientific and Cell Signaling Technology, respectively.

### HPRT enzymatic assay

HPRT enzyme activity was determined as previously described [6]. The IMP and AMP positions scraped from the TLC plates were mixed with Insta-Gel Plus liquid scintillation cocktail (PerkinElmer, Waltham, MA) and radioactivity was measured using a Beckman Coulter LS 6500 liquid scintillating counter (Brea, CA).

## Supporting Information

File S1 **Supplementary Tables** **S1, S2, S3, and S4.**
(DOCX)

## References

[1] Jinnah HA, Friedmann T (2000) Lesch-Nyhan Disease and its Variants. Metabolic and Molecular Bases of Inherited Disease Scriver, Beaudet, Sly and Valle (eds.), McGraw Hill 2537–2570.

[2] Yeh J, Zheng S, Howard BD (1998) Impaired differentiation of HPRT-deficient dopaminergic neurons: a possible mechanism underlying neuronal dysfunction in Lesch-Nyhan syndrome. J Neurosci Res 53(1): 78–85. 9670994 10.1002/(SICI)1097-4547(19980701)53:1<78::AID-JNR8>3.0.CO;2-G

[3] Smith DW, Friedmann T (2000) Characterization of the dopamine defect in primary cultures of dopaminergic neurons from hypoxanthine phosphoribosyltransferase knockout mice. Mol Ther 1: 486–491. 10933970 10.1006/mthe.2000.0057

[4] Ceballos-Picot I, Mockel L, Potier MD, Dauphinot L, Shirley TL, et al (2009) Hypoxanthine-guanine phosphoribosyl transferase regulates early developmental programming of dopamine neurons: implications for Lesch-Nyhan disease pathogenesis. Hum Mol Genet 18: 2317–2327. 19342420 10.1093/hmg/ddp164 PMC2694685

[5] Guibinga GH, Hsu S, Friedmann T (2010) Deficiency of the housekeeping gene hypoxanthine-guanine phosphoribosyltransferase (HPRT) dysregulates neurogenesis. Mol Ther 18: 54–62. 19672249 10.1038/mt.2009.178 PMC2839227

[6] Kang TH, Guibinga G, Jinnah HA, Friedmann T (2011) HPRT deficiency coordinately dysregulates canonical WNT and presenilin-1 signaling: a neuro-developmental regulatory role for a housekeeping gene? PLoS One 6(1): e16572 10.1371/journal.pone.oo16572 21305049 PMC3030599

[7] Mastrangelo L, Kim JE, Miyanohara A, Kang TH, Friedmann T (2012) Purinergic signaling in human pluripotent cells is regulated by the housekeeping gene hypoxanthine guanine phosphoribosyltransferase. Proc Natl Acad Sci USA 109(9): 3377–3382. 22331909 10.1073/pnas.1118067109 PMC3295269

[8] Guibinga GH, Hrustanovic G, Bouic K, Jinnah HA, Friedmann T (2012) MicroRNA-mediated dysregulation of neural developmental genes in HPRT deficiency: Clues for Lesch Nyhan disease? Hum Mol Genet 21(3): 609–622. 22042773 10.1093/hmg/ddr495 PMC3259014

[9] Cho MS, Hwang DY, Kim DW (2008) Efficient derivation of functional dopaminergic neurons from human embryonic stem cells on a large scale. Nat Protoc 3(12): 1888–1894. 19008875 10.1038/nprot.2008.188

[10] Visser JE, Bär PR, Jinnah HA (2000) Lesch-Nyhan disease and the basal ganglia. Brain Res Brain Res Rev 32(2–3): 449–475. 10760551 10.1016/s0165-0173(99)00094-6

[11] Jinnah HA, Wojcik BE, Hunt M, Narang N, Lee KY, Goldstein M, et al (1994) Dopamine deficiency in a genetic mouse model of Lesch-Nyhan disease. J Neurosci 3(Pt 1): 1164–1175. 10.1523/JNEUROSCI.14-03-01164.1994 PMC6577527 7509865

[12] Egami K, Yitta S, Kasim S, Lewers JC, Roberts RC, et al (2007) Basal ganglia dopamine loss due to defect in purine recycling. Neurobiol Dis 26(2): 396–407. 17374562 10.1016/j.nbd.2007.01.010 PMC1930158

[13] Deutsch SI, Long KD, Rosse RB, Mastropaolo J, Eller J (2005) Hypothesized deficiency of guanine-based purines may contribute to abnormalities of neurodevelopment, neuromodulation, and neurotransmission in Lesch-Nyhan syndrome. Clin Neuropharmacol 28(1): 28–37. 15711436 10.1097/01.wnf.0000152043.36198.25

[14] Liberzon A, Subramanian A, Pinchback R, Thorvaldsdóttir H, Tamayo P, et al (2011) Molecular signatures database (MSigDB) 3.0. Bioinformatics 27(12): 1739–1740 10.1093/bioinformatics/btr260 21546393 PMC3106198

[15] Irizarry RA, Wang C, Zhou Y, Speed TP (2009) Gene set enrichment analysis made simple. Stat Methods Med Res 18(6): 565–575 10.1177/0962280209351908 20048385 PMC3134237

[16] Krady JK, Basu A, Levison SW, Milner RJ (2002) Differential expression of protein tyrosine kinase genes during microglial activation. Glia 40(1): 11–24. 12237840 10.1002/glia.10101

[17] Suh HS, Kim MO, Lee SC (2005) Inhibition of granulocyte-macrophage colony-stimulating factor signaling and microglial proliferation by anti-CD45RO: role of Hck tyrosine kinase and phosphatidylinositol 3-kinase/Akt J Immunol. 174(5): 2712–2719. 10.4049/jimmunol.174.5.2712 15728479

[18] Sandhoff K, Kolter T (1998) Processing of sphingolipid activator proteins and the topology of lysosomal digestion. Acta Biochim Pol 45(2): 373–384. 9821868

[19] Saxena M, Williams S, Brockdorff J, Gilman J, Mustelin T (1999) Inhibition of T cell signaling by mitogen-activated protein kinase-targeted hematopoietic tyrosine phosphatase (HePTP). J Biol Chem 274(17): 11693–11700. 10206983 10.1074/jbc.274.17.11693

[20] Saxena M, Williams S, Taskén K, Mustelin T (1999) Crosstalk between cAMP-dependent kinase and MAP kinase through a protein tyrosine phosphatase. Nat Cell Biol 1(5): 305–311. 10559944 10.1038/13024

[21] Chen G, Gharib TG, Huang CC, Taylor JM, Misek DE, et al (2002) Discordant protein and mRNA expression in lung adenocarcinomas. Mol Cell Proteomics Apr 1(4): 304–13. 10.1074/mcp.m200008-mcp200 12096112

[22] Pascal LE, True LD, Campbell DS, Deutsch EW, Risk M, et al (2008) Correlation of mRNA and protein levels: cell type-specific gene expression of cluster designation antigens in the prostate. BMC Genomics 2008 May 23 9: 246 10.1186/1471-2164-9-246 PMC2413246 18501003

[23] László A, Vörös E, Buga K, Horváth K, Mayer P, et al (2009) Myelination disturbance in a patient with hyperuricemia and hyperserotoninemia combined with 18q deletion syndrome. Ideggyogy Sz 62(11–12): 413–417. 20025132

[24] Makoukji J, Shackleford G, Meffre D, Grenier J, Liere P, et al (2011) Interplay between LXR and Wnt/ß-catenin signaling in the negative regulation of peripheral myelin genes by oxysterols. J Neurosci 31(26): 9620–9629 10.1523/JNEUROSCI.0761-11.2011 21715627 PMC6623163

[25] Tawk M, Makoukji J, Belle M, Fonte C, Trousson A, et al (2011) Wnt/beta-catenin signaling is an essential and direct driver of myelin gene expression and myelinogenesis. J Neurosci 31(10): 3729–3742 10.1523/JNEUROSCI.4270-10.2011 21389228 PMC6622795

[26] Azim K, Butt AM (2011) GSK3ß negatively regulates oligodendrocyte differentiation and myelination in vivo. Glia 59(4): 540–553 10.1002/glia.21122 21319221

[27] Li H, Richardson WD (2009) Genetics meets epigenetics: HDACs and Wnt signaling in myelin development and regeneration. Nat Neurosci 12(7): 815–817. 19554044 10.1038/nn0709-815

[28] Lewallen KA, Shen YA, De la Torre AR, Ng BK, Meijer D, et al (2011) Assessing the role of the cadherin/catenin complex at the Schwann cell-axon interface and in the initiation of myelination. J Neurosci 31(8): 3032–3043 10.1523/JNEUROSCI.4345-10.2011 21414924 PMC3758556

[29] Feigenson K, Reid M, See J, Crenshaw EB 3rd, Grinspan JB (2009) Wnt signaling is sufficient to perturb oligodendrocyte maturation. Mol Cell Neurosci 42(3): 255–265. 19619658 10.1016/j.mcn.2009.07.010

[30] Jacob C, Christen CN, Pereira JA, Somandin C, Baggiolini A, et al (2011) HDAC1 and HDAC2 control the transcriptional program of myelination and the survival of Schwann cells. Nat Neurosci 14(4): 429–436 10.1038/nn.2762 21423190

[31] Chen Y, Wang H, Yoon SO, Xu X, Hottiger MO, et al (2011) HDAC-mediated deacetylation of NF-κB is critical for Schwann cell myelination. Nat Neurosci 14(4): 437–441 10.1038/nn.2780 21423191 PMC3074381

[32] Schebesta M, Serluca FC (2009) Olig1 expression identifies developing oligodendrocytes in zebrafish and requires hedgehog and notch signaling. Dev Dyn 238(4): 887–898. 19253391 10.1002/dvdy.21909

[33] Park HC, Mehta A, Richardson JS, Appel B (2002) Olig2 is required for zebrafish primary motor neuron and oligodendrocyte development. Dev Biol 248(2): 356–368. 12167410 10.1006/dbio.2002.0738

[34] Wang SZ, Dulin J, Wu H, Hurlock E, Lee SE, et al (2006) An oligodendrocyte-specific zinc-finger transcription regulator cooperates with Olig2 to promote oligodendrocyte differentiation. Development 133(17): 3389–3398. 16908628 10.1242/dev.02522

[35] Nicolay DJ, Doucette JR, Nazarali AJ (2007) Transcriptional control of oligodendrogenesis. Glia 55(13): 1287–1299. 17647291 10.1002/glia.20540

[36] Park HC, Appel B (2003) Delta-Notch signaling regulates oligodendrocyte specification. Development 130(16): 3747–3755. 12835391 10.1242/dev.00576

[37] Wang Z, Oron E, Nelson B, Razis S, Ivanova N (2012) Distinct lineage specification roles for NANOG, OCT4, and SOX2 in human embryonic stem cells. Cell Stem Cell 10(4): 440–454. 22482508 10.1016/j.stem.2012.02.016

[38] Graham V, Khudyakov J, Ellis P, Pevny L (2003) SOX2 functions to maintain neural progenitor identity. Neuron 39(5): 749–765. 12948443 10.1016/s0896-6273(03)00497-5

[39] Bylund M, Andersson E, Novitch BG, Muhr J (2003) Vertebrate neurogenesis is counteracted by Sox1-3 activity. Nat Neurosci 6(11): 1162–1168. 14517545 10.1038/nn1131

[40] Ellis P, Fagan BM, Magness ST, Hutton S, Taranova O, et al (2004) SOX2, a persistent marker for multipotential neural stem cells derived from embryonic stem cells, the embryo or the adult. Dev Neurosci 26(2–4): 148–165. 15711057 10.1159/000082134

[41] Papanayotou C, Mey A, Birot AM, Saka Y, Boast S, et al (2008) A mechanism regulating the onset of Sox2 expression in the embryonic neural plate. PLoS Biol 6(1): e2. 10.1371/journal.pbio.0060002 PMC2174969 18184035

[42] Bani-Yaghoub M, Tremblay RG, Lei JX, Zhang D, Zurakowski B, et al (2006) Role of Sox2 in the development of the mouse neocortex. Dev Biol 295(1): 52–66. 16631155 10.1016/j.ydbio.2006.03.007

[43] Evsen L, Sugahara S, Uchikawa M, Kondoh H, Wu DK (2013) Progression of neurogenesis in the inner ear requires inhibition of sox2 transcription by neurogenin1 and neurod1. J Neurosci 33(9): 3879–3890 10.1523/JNEUROSCI.4030-12.2013 23447599 PMC3865497

[44] Deutsch SI, Long KD, Rosse RB, Mastropaolo J, Eller J (2005) Hypothesized deficiency of guanine-based purines may contribute to abnormalities of neurodevelopment, neuromodulation, and neurotransmission in Lesch-Nyhan syndrome. Clin Neuropharmacol 28(1): 28–37. 15711436 10.1097/01.wnf.0000152043.36198.25

[45] Pinto CS, Seifert R (2006) Decreased GTP-stimulated adenylyl cyclase activity in HPRT-deficient human and mouse fibroblast and rat B103 neuroblastoma cell membranes. J Neurochem 96(2): 454–459. 16336632 10.1111/j.1471-4159.2005.03570.x

[46] Pinto CS, Jinnah HA, Shirley TL, Nyhan WL, Seifert R (2005) Altered membrane NTPase activity in Lesch-Nyhan disease fibroblasts: comparison with HPRT knockout mice and HPRT-deficient cell lines. J Neurochem 93(6): 1579–1586. 15935074 10.1111/j.1471-4159.2005.03151.x

[47] Garcia M, Puig J, Torres R (2012) Adenosine, dopamine and serotonin receptors imbalance in lymphocytes of Lesch-Nyhan patients. Journal of Inherited Metabolic Disease 35(6): 1129–1135. 22403020 10.1007/s10545-012-9470-5

[48] Bertelli M, Alushi B, Veicsteinas A, Jinnah HA, Micheli V (2009) Gene expression and mRNA editing of serotonin receptor 2C in brains of HPRT gene knock-out mice, an animal model of Lesch-Nyhan disease. J Clin Neurosci 16(8): 1061–1063 10.1016/j.jocn.2008.12.011 19473847 PMC4871153

[49] Bertelli M, Cecchin S, Lapucci C, Jacomelli G, Jinnah HA, et al (2006) Study of the adenosinergic system in the brain of HPRT knockout mouse (Lesch-Nyhan disease). Clin Chim Acta 373(1–2): 104–107. 16793031 10.1016/j.cca.2006.05.013

[50] Saito Y, Takashima S (2000) Neurotransmitter changes in the pathophysiology of Lesch-Nyhan syndrome. Brain Dev 22 (Suppl. 1)S122–S131. 10984673 10.1016/s0387-7604(00)00143-1

[51] Esposito G, Scuderi C, Valenza M, Togna GI, Latina V, et al (2011) Cannabidiol reduces Aß-induced neuroinflammation and promotes hippocampal neurogenesis through PPARγ involvement. PLoS One 6(12): e28668 10.1371/journal.pone.0028668 22163051 PMC3230631

[52] Sadeghian M, Marinova-Mutafchieva L, Broom L, Davis JB, Virley D, et al (2012) Full and partial peroxisome proliferation-activated receptor-γ agonists, but not δ agonist, rescue of dopaminergic neurons in the 6-OHDA parkinsonian model is associated with inhibition of microglial activation and MMP expression. J Neuroimmunol 246(1–2): 69–77. 22498097 10.1016/j.jneuroim.2012.03.010

[53] Edgar R, Domrachev M, Lash AE (2002) Gene Expression Omnibus: NCBI gene expression and hybridization array data repository. Nucleic Acids Res 30(1): 207–210. 11752295 10.1093/nar/30.1.207 PMC99122

[54] Trapnell C, Pachter L, Salzberg SL (2009) TopHat: discovering splice junctions with RNA-Seq. Bioinformatics 25(9): 1105–1111 10.1093/bioinformatics/btp120 19289445 PMC2672628

[55] Trapnell C, Williams BA, Pertea G, Mortazavi A, Kwan G, et al (2010) Transcript assembly and quantification by RNA-Seq reveals unannotated transcripts and isoform switching during cell differentiation. Nat Biotechnol 28(5): 511–515 10.1038/nbt.1621 20436464 PMC3146043

[56] Trapnell C, Hendrickson DG, Sauvageau M, Goff L, Rinn JL, et al (2013) Differential analysis of gene regulation at transcript resolution with RNA-seq. Nat Biotechnol 31(1): 46–53 10.1038/nbt.2450 23222703 PMC3869392

