# Supplementary figures and images for: The Housekeeping Gene Hypoxanthine Guanine Phosphoribosyltransferase (HPRT) Regulates Multiple Developmental and Metabolic Pathways of Murine Embryonic Stem Cell Neuronal Differentiation
Source: PLoS One. 2013 Oct 9;8(10):e74967. doi: 10.1371/journal.pone.0074967 (PMC3794013; doi:10.1371/journal.pone.0074967)

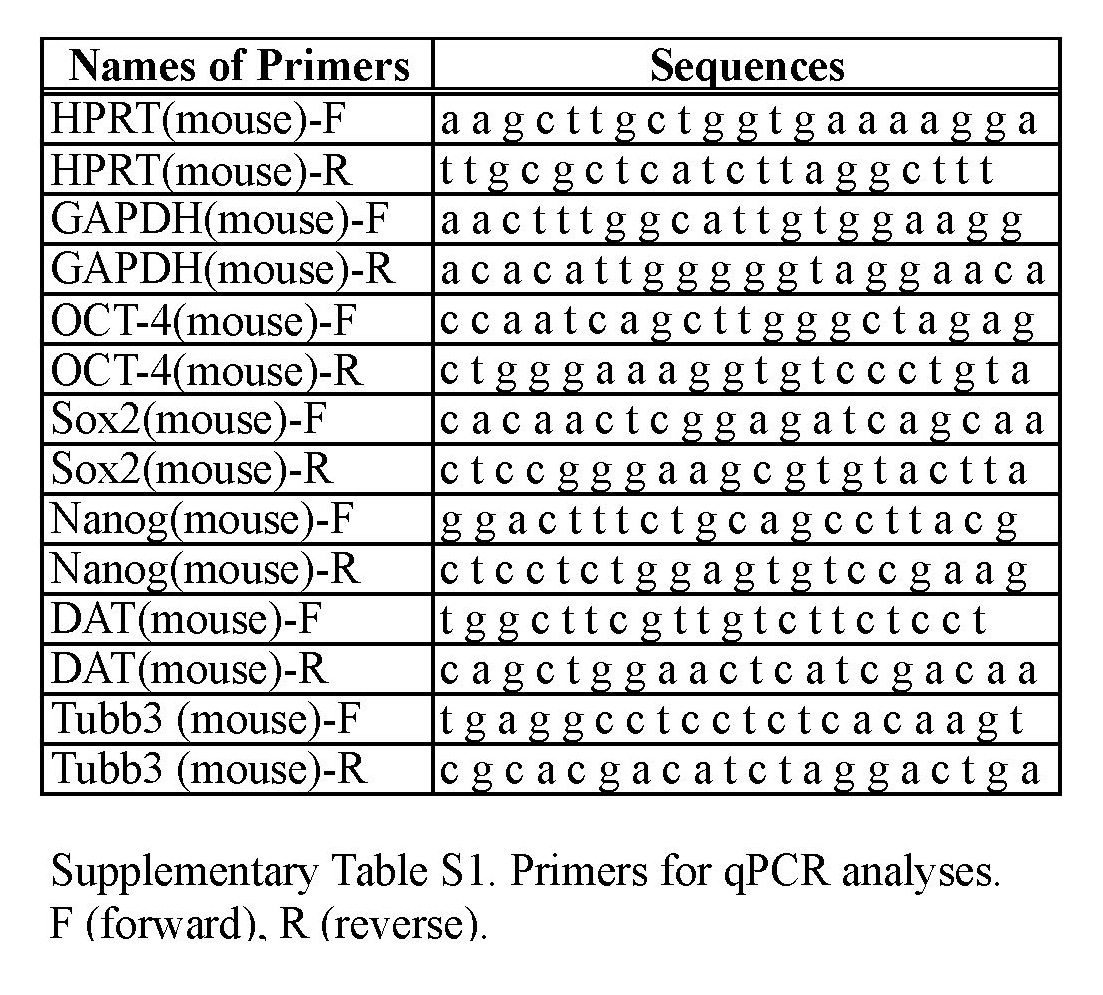


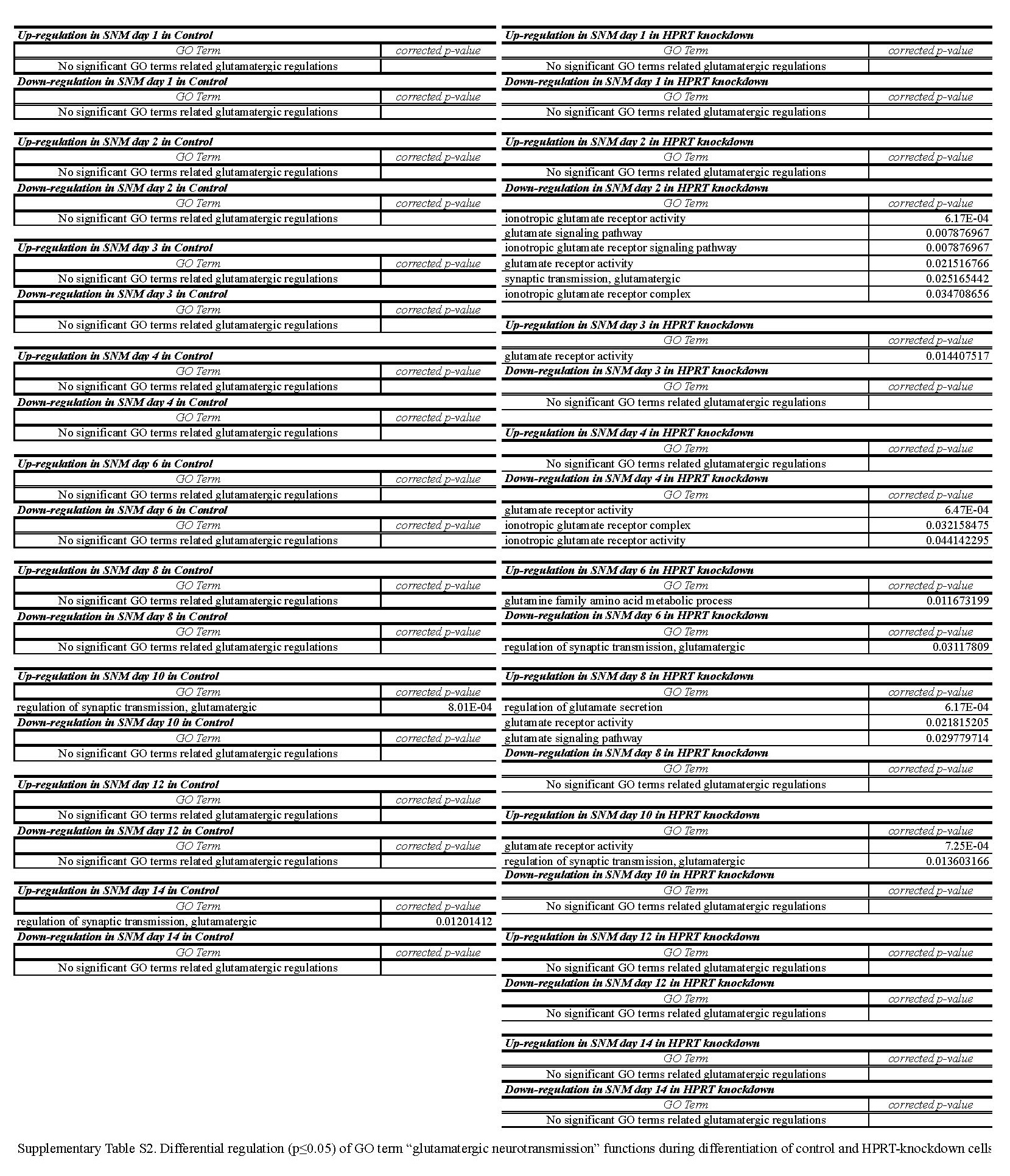


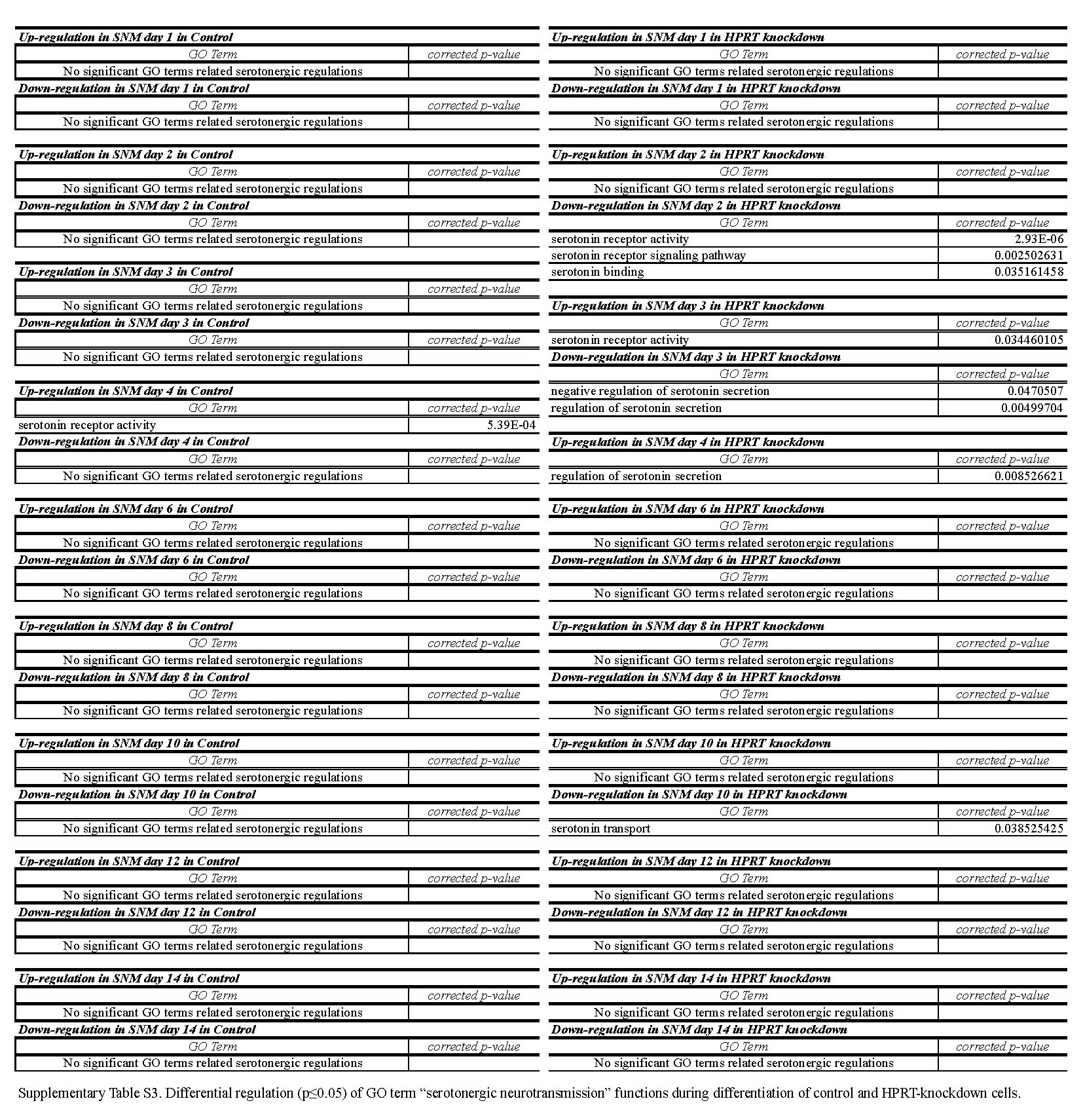


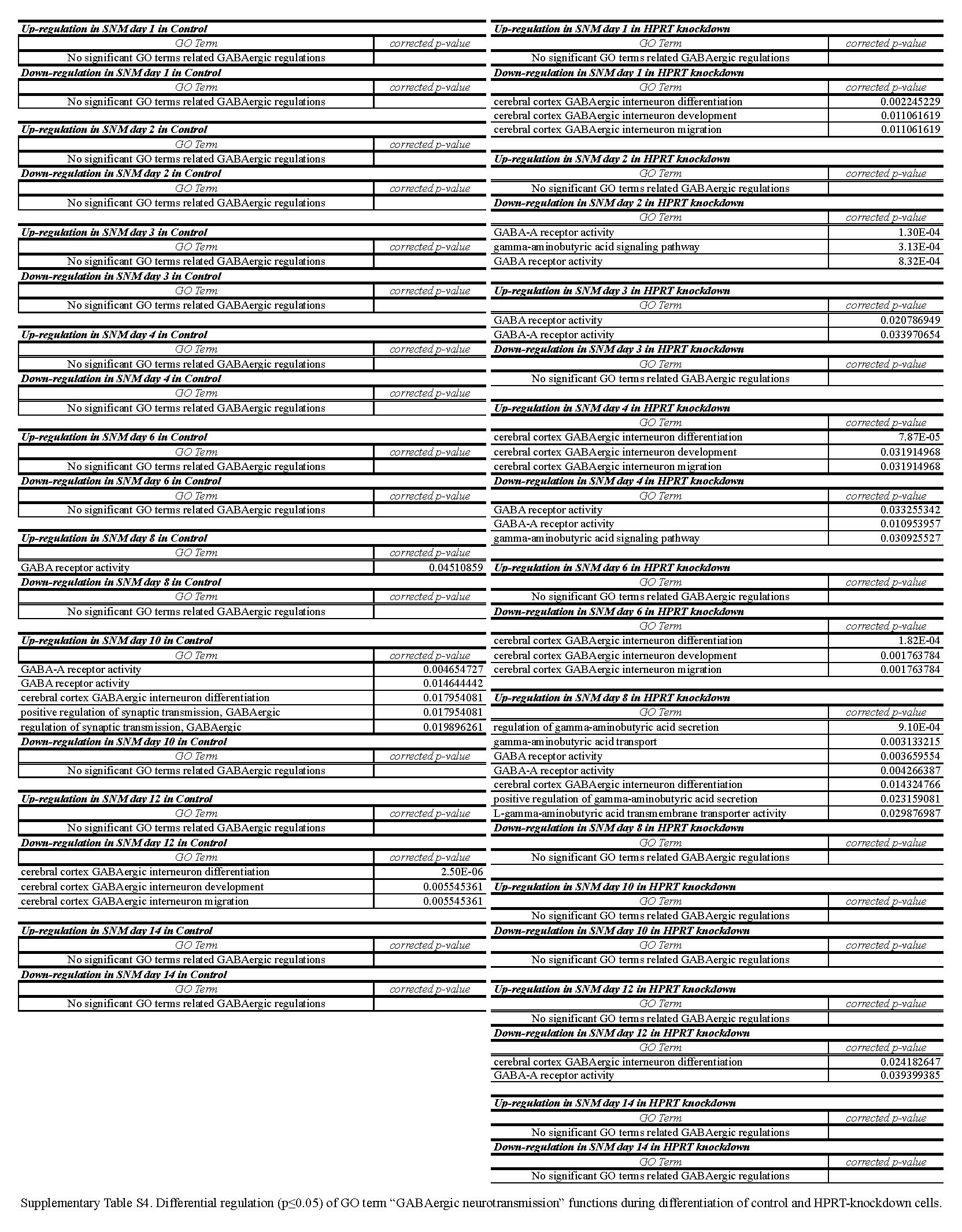

Supplement: File S1 — Supplementary Tables S1, S2, S3, and S4. (DOCX) [file pone.0074967.s001.docx]
